# Risk of Subsequent Hysterectomy after Expectant Management in the Treatment of Placenta Accreta Spectrum Disorders

**DOI:** 10.3390/medicina58050678

**Published:** 2022-05-19

**Authors:** Anca Maria Panaitescu, Gheorghe Peltecu, Radu Botezatu, George Iancu, Nicolae Gica

**Affiliations:** 1 Department of Obstetrics and Gynecology, Carol Davila University of Medicine and Pharmacy, 020021 Bucharest, Romania; gheorghe.peltecu@umfcd.ro (G.P.); radu.botezatu@umfcd.ro (R.B.); george.iancu@umfcd.ro (G.I.); gica.nicolae@umfcd.ro (N.G.); 2 Filantropia Clinical Hospital, 011171 Bucharest, Romania

**Keywords:** placenta accreta spectrum (PAS), diagnosis, classification, expectant management

## Abstract

Management strategies for pregnancies with abnormal adherence/invasion of the placenta (placenta accreta spectrum, PAS) vary between centers. Expectant management (EM), defined as leaving the placenta in situ after the delivery of the baby, until its complete decomposition and elimination, has become a potential option for PAS disorders in selected cases, in which the risk of Caesarean hysterectomy is very high. However, expectant management has its own risks and complications. The aim of this study was to describe the rates of subsequent hysterectomy (HT) in patients that underwent EM for the treatment of PAS disorders. We reviewed the literature on the subject and found 12 studies reporting cases of HT after initial intended EM. The studies included 1918 pregnant women diagnosed with PAS, of whom 518 (27.1%) underwent EM. Out of these, 121 (33.2%) required subsequent HT in the 12 months following delivery. The rates of HT after initial EM were very different between the studies, ranging from 0 to 85.7%, reflecting the different characteristics of the patients and different institutional management protocols. Prospective multicenter studies, in which the inclusion criteria and management strategies would be uniform, are needed to better understand the role EM might play in the treatment of PAS disorders.

## 1. Introduction

In 1937, Irving and Hertig were the first to publish a cohort study including 20 cases of placenta accreta and a review on the previous 86 cases reported in the literature at that time [1]. All the cases were described as abnormal adherence of the placenta to the uterine wall, completely or partially, with the absence of decidua basalis in the area of adhesion. Among the possible etiologic factors mentioned were the manual removal of the placenta or uterine curettage, as only one case out of the 20 included in the cohort had a previous Caesarean section. In 1966, Luke et al. described placenta accreta as a spectrum of abnormal placentation with various degrees of invasion, from placenta vera or creta to placenta increta or percreta [2]. In 2018, the International Federation of Obstetrics and Gynecology (FIGO) proposed new terminology that included all three grades of abnormal placentation under the acronym of PAS (placenta accreta spectrum) [3], subsequently proposing a new clinical classification of PAS in three grades [4].

The incidence of PAS disorders in the general population of pregnant women is estimated at 1.7/10,000 pregnancies [5,6] and has been growing steadily over time, probably in relation to the increasing rates of Caesarean delivery [7,8,9].

Ultrasound is an excellent tool for the prenatal diagnosis of PAS [10] and, although some guidelines do not recommend a generalized screening program [11], the previous obstetrical history, with a clear recognition of the risk factors, and the systematic ultrasound evaluation of high-risk patients play an important role in antenatal diagnosis and allow planned intervention with a multidisciplinary approach.

There are three important stages in the care of pregnant women at increased risk of PAS: the recognition of the risk factors, establishing an accurate prenatal diagnosis, and referral to a tertiary center with expertise for follow-up and delivery [12]. The diagnosis of PAS is confirmed clinically, during CS, when, after the delivery of the baby, the placenta does not detach. In this situation, when an elective Caesarean hysterectomy (CSHT) can be safely performed, the obstetrician starts the procedure. This is considered the gold standard of treatment by many. There are some cases, however, in which the risk of bleeding and/or vesical-ureteral injuries are high, and the obstetrician can decide to leave the placenta in situ, ligate the umbilical cord close to placental insertion, and close the uterus and abdominal wall for a delayed hysterectomy (DHT), which is planned for 4–6 weeks later, or for expectant management (EM). Delayed hysterectomy, a hybrid strategy, is aimed at minimizing blood loss and avoiding visceral lesions, especially of the bladder and ureters [13]. Expectant management represents a real conservative option and is achieved by leaving the placenta in situ until its complete decomposition and elimination, with the intention of preventing heavy bleeding and reducing the risk of severe maternal complications related to post-Caesarean hemostasis hysterectomy [14]. Expectant management is preferred when intraoperative findings suggest an unacceptably high risk of bleeding or urinary tract injuries, or when the patient desires future fertility [14,15,16]. Another conservative management strategy consists in placental-myometrial en bloc resection and repair, described by Palacios et al. [17], or triple P procedure, described by Chandraharan et al., as an alternative to peri-partum hysterectomy or conservative management by retaining the placenta [18]. Interventional radiology techniques, such as pelvic artery embolization, can sometimes be used as adjuvants to any of these strategies in order to limit blood loss [19].

The aim of our study was to review the recent literature published on the method of EM in treating PAS and to describe the risk of subsequent hysterectomy when this approach is used. We also aimed to underline the possible advantages and disadvantages of the different modalities employed in the treatment of PAS.

## 2. Materials and Methods

We undertook an analysis of the reviews and single/multicenter studies that report maternal outcomes, and specifically, the rate of subsequent HT, when EM is used for the treatment of PAS disorders. PubMed was searched for studies published from the beginning of records in relation to treatment of PAS disorders. We then selected those dealing with expectant management and that were published in the last 15 years. We searched for the terms “expectant”, “expectative”, or “conservative” and “placenta accreta”, “percreta”, and “increta”. The search retrieved 468 entries. We selected the studies and literature reviews that included more than three reported cases.

## 3. Results

We found 12 studies that met our search criteria, published between 2010 and 2022, that included cases of PAS managed expectantly. Of the twelve studies (Table 1), nine had a retrospective design, while three had a prospective approach to enrolling patients. The 12 studies included 1918 cases of pregnant women diagnosed with PAS disorders, of whom 518 (27.1%) were expectantly managed, which was defined as “leaving the placenta in situ”. Of those initially managed by EM, 121 (33.2%) required subsequent hysterectomy up to 12 months following delivery. Table 1 summarizes the main findings of the studies.

We found large variations in the reported rates of subsequent HT between the studies in the centers where EM was used for the treatment of PAS. Most studies were retrospective, inherently presenting specific biases. Patients with different severities of PAS were included: in some of the studies, there were differences in the severity of PAS based on the prenatal diagnosis (percreta, accreta/increta), while many of the studies did not report a PAS grade. In some of the studies (Table 1), there was an intention to remove all the placenta, or parts of it, before EM was offered. We also noticed from the analyzed studies that during the process of EM, other strategies, such as interventional radiology techniques were added to improve success.

## 4. Discussion

**Figure 1:**
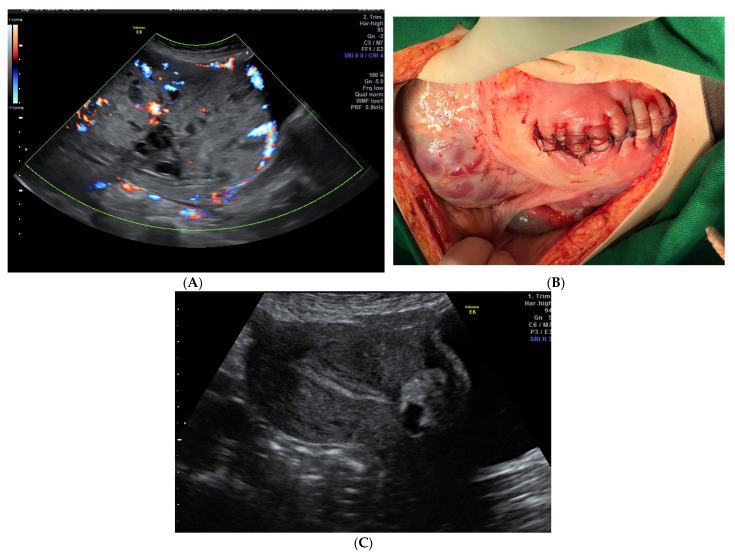
(**A**). Ultrasound appearance of the placenta at 35 weeks of pregnancy. (**B**). Placenta left in situ and corporeal-fundal uterine suture at delivery. (**C**). Transabdominal ultrasound examination 27 weeks after delivery. On the anterior uterine wall, the small placenta is noted, and the endometrium is linear. The patient had a history of CS and, at 20 weeks, she was diagnosed with placenta previa with a high suspicion of anterior abnormal invasion to the urinary bladder. She had no vaginal bleeding. Planned Caesarean section was performed at 36 weeks of gestation, with a vertical mid-line incision chosen for the abdomen and a fundal incision of the uterus to avoid the upper pole of the placenta. After the delivery of the baby, the decision to leave the placenta in situ was taken to avoid significant bleeding and bladder injury. After the ligature of the umbilical cord close to its placental insertion, the uterine wall was sutured (**B**). Close monitoring was offered and, at 27 weeks after delivery, the placenta was almost fully evacuated (**C**) and the patient had normal menstruation.

There is a lack of consensus regarding the optimal strategy for the management of PAS disorders [16], with a wide variation around the globe [32,33]. When PAS is suspected before delivery, the patient/couple should be counseled and involved in the decisions over treatment. A question that needs to be answered when offering the option of EM when treating PAS is related to the risks of complications and need for a subsequent HT. Furthermore, women managed expectantly should be informed that they require long-term follow-up, with multiple hospital visits for blood tests and ultrasounds (Figure 1) and immediate access to health care facilities [34].

The treatment options for PAS and their advantages and disadvantages are presented in Table 2.

Our study aimed to review the outcomes of EM for PAS, especially in relation to the need for subsequent hysterectomy. Overall, in the studies included in our paper, 33.2% of women with PAS disorders who underwent EM required HT. The rate of success of EM observed in our study (67.8%) is similar to the figures published recently in a review by Sentilhes et al. [33], who found that the uterine preservation rate with EM was 78%, and that the rate of severe maternal morbidity was about 6%. A summary of other reviews published in the literature on the subject is presented in Table 3.

The overall need for HT in those who underwent initial expectant management was 37.5% in the reviews included. Again, the rates of reported HT were different between the studies. The reviews reported the complications that can arise during EM as hemorrhage, infection, fistulas and sepsis, coagulopathy, and even maternal death. There is a lack of consensus on how expectant management for treating PAS is defined in the literature, who should benefit from this approach, and how follow-ups should be arranged. The primary and secondary outcomes reported by different studies are very heterogenous (obtaining statistical power to test a hypothesis requires a large number of participants, or PAS disorders are relatively rare). Our paper reflects these large differences between studies. Another issue regarding the expectant management of PAS disorders is the lack of histological confirmation of the diagnosis. When a hysterectomy is performed for PAS, specimens can be examined fresh by a senior pathologist, together with the lead obstetrician, to establish the existence of an abnormal adhesion, complete or focal. Biopsies are taken from the most suspicious areas. Correct diagnosis and reporting allow a better correlation with the US aspects and a possible reassessment of future management [39]. Currently, the diagnosis of placenta with abnormal adhesion is dominated by clinics, which leads to overdiagnosis [40]. Histopathology confirmation should become the gold standard for diagnosing PAS [39]. A correct histopathological diagnosis and the adoption of FIGO classification criteria also allow a reassessment of the epidemiological data [40]. Unfortunately, the histological diagnosis cannot be obtained in case of the expectant management of PAS [41].

Our study has certain limitations that we acknowledge. We did not have the required data to undertake a systematic review related to the subject; thus, there may have been subjectivity bias. We included studies published in the English and French languages, potentially missing reports from different settings in low- or middle-income countries. Nevertheless, our study could be of benefit to doctors counseling women with PAS regarding options for management and their associated risk. It is surprising that the results of the EM were so scattered and so different between the centers. Future prospective multicenter studies are required to better understand the role of expectant management in the treatment of PAS. These studies should be controlled and include centers with similar management protocols. The most convincing results would be achieved through a randomized controlled trial, in which EM could be compared with other strategies, if feasible. Regarding counseling, a more pragmatic and approach, focused on providing patients with information, would be for centers treating PAS disorders to audit their own data and to offer counseling based on these data.

## 5. Conclusions

Although Caesarean hysterectomy is recommended by most authorities as the gold standard, the expectant management of PAS disorders is increasingly used with the goals of avoiding severe maternal morbidity, or even mortality, associated with surgery, as well as preserving fertility. There is a growing body of evidence showing that expectant management, in selected and carefully monitored cases, could be successful. In our study, approximately 1 in 3 women required a HT after expectant management. When complications occur during follow-up for EM, they can potentially be treated with fewer risks, by multidisciplinary teams, in dedicated centers. There are limited data on expectant management in placenta percreta. The application of expectant management in PAS disorders has the disadvantage of the lack of histopathological diagnosis.

We acknowledge that PAS disorders are iatrogenic pathologies and, despite encouraging success, expectant management is still a debated subject. A safer reduction in the rate of primary Caesarean delivery would reduce the incidence of and risks associated with PAS disorders. Pregnant women, especially in countries with a high rate of CSs, doctors, and society at large, should be aware of the potential complications associated with CS.

## Figures and Tables

**Table 1:** Studies in which expectant management was intended.

Author, Year	Study Design	Nr. Cases	PAS Preop. Diagnosis *		Management		EM Definition	HT After EM *n*/total EM (%)	Comments
			A/I	P	CSHT	EM			
Marcellin et al., 2018 [20]	Retrospective, France	156		51	P: 27/51 (52.9%)	P: 24/51 (47%)	Leaving the placenta in situ	17/24 (70.8%)	Indications for HT: - septic shock - hemorrhage Other complications: - bladder injury - vesical-vaginal fistula - ureteral injury
			105		A/I: 22/105 (20.9%)	A/I: 83/105 (79%)	Leaving the placenta in situ	4/83 (4.8%)	
Daney de Marcillac et al., 2016 [21]	Retrospective, France	15				15	Leaving the placenta totally in situ	3/15 (20%)	In the other 12 with EM: - one case had hemorrhage managed with embolization and four had endometritis managed with antibiotics
Sentilhes et al., 2010 [22]	Retrospective multicenter	167	+		18	149	Placenta left in situ, partially or totally, with no attempt to remove it forcibly	18/149 (12.1%)	There were 10 cases (6%) with: sepsis septic shock fistula DVT peritonitist
Fitzpatrick et al., 2014 [23]	Population-based descriptive, UK	134	+	+	118	16	No attempt to remove the placenta	5/16 (31.3%)	From the 16 cases with EM: - HT in 5 cases
Sentilhes et al., 2021 [24]	Prospective, observational cohort	148	+	+	62	86	Obstetrician’s decision to leave the placenta partially or totally in situ	19/86 (22.1%)	Of the 86 cases with EM: - 19 HT - 21 embolization - 9 endometritis - 24 readmissions <6 months
Bassetty et al., 2021 [25], India	Retrospective observational	21	+	+	17	4		0/4 (0%)	Additional methods used to EM: - one bilateral uterine artery ligation; - two UAE
van Beekhuizen. et al., 2021 [26]	Observational multicenter study	442	+	+	252	48	Placenta was intentionally left in situ	20/48 (41.6%)	In 90, placenta detached at delivery; the others were managed by other methods;
Lional et al., 2021 [27]	Single-center retrospective cohort study, Singapore	90	+	+	51	23		9/23 (39.1%)	Other management types in 16
Chevalier et al., 2020 [28]	Single-center retrospective study, France	46	+	+	34	12		8/12 (66.6%)	
Miyakoshi et al., 2018 [29]	Retrospective, multicenter study, Japan	613				36	Placenta left in situ	11/36 (30.5%)	
Kutuk et al., 2017 [30]	Retrospective single-center cohort study, Turkey	79	+	+	27	15		1/15 (6.66%)	Other conservative management types in 37
Su et al., 2017 [31]	Single-center retrospective study, Taiwan	7	+	+		7	Placenta left in situ	6/7 (85.7%)	

PAS: placenta accrete spectrum; A: accreta; I: increta; P: percreta; CSHT: Caesarean hysterectomy; EM: expectant management; UAE: uterine artery embolization; DVT: deep-vein thrombosis. *: Imaging, clinical, histopathology diagnosis.

**Table 2:** Analysis of different management decisions in PAS disorders.

	Advantages	Disadvantages	Comments
Primary Hysterectomy (Caesarean hysterectomy)	- Standard procedure - When technically feasible, no risk associated to follow-up when compared to DHT or EM	- Does not preserve fertility - Risk of massive bleeding - Related organ injuries - Maternal death.	Severe morbidity associated with increasing severity of PAS
Delayed planned Hysterectomy after leaving placenta in situ	- Decision based on estimation of resectability - Less blood loss - Less transfusion	- Risk of bleeding, infection, DIC, pulmonary embolism	Adequate strategy for settings where complex surgical procedures cannot be undertaken in an emergency Planed DHT at 4–6 weeks postpartum Better surgical conditions
Expectant management	- Prevents massive bleeding and urinary tract injuries - Preserves fertility - Reduces transfusion rate at the time of surgery	- Risk of bleeding, sepsis, DIC, pulmonary embolism, renal failure, fistula, maternal death	Long-term follow-up Can become an emergency

DIC: disseminated intravascular coagulation; DHT: delayed hysterectomy.

**Table 3:** Review studies on expectant management in PAS.

Author, Year	Nr. Cases	Management		Definition of EM	HT after EM	Composite Maternal Morbidity After Expectant Management
		CHT	EM			
Clausen, 2014 [35]	119		36	“placenta left in situ”	21/36 (58.3%)	From the 36 cases: - Late complications: 61% - bladder injury: 11 cases - postop hemorrhage: 5 - fistula: 2 - pulmonary embolism: 1 - Early complications: 12 % - Planned HT: 3
Pather, 2014 [36]	57	10	47		23/40 (57.5%)	In the 47 cases: - Late complications (42%) - Sepsis: 4 cases - DIC: 6 cases - Fistula: 1 case - PPH: 11 cases
Steins Bisschop, 2011 [37]	295		287		55/287 (19.1%)	Secondary HT: 55/287 (19%) 1 maternal death **
Timmermans, 2007 [38]	60	44; other types of conservative management (medical/radiological)	26	Management without additional interventions	4/26 (15.3%)	Cases: - infection: 11 cases/60 - vaginal bleeding: 21 cases/60 - DIC: 4 cases/60

PAS: placenta accrete spectrum; EM: expectant management; DIC: disseminated intravascular coagulation; PPH: postpartum hemorrhage. **: due to myelosuppression and nephrotoxicity secondary to methotrexate injection into the umbilical cord.
